# Association of Different Types of Diabetic Autonomic Neuropathy With Left Ventricular Diastolic Dysfunction in Patients With Type 2 Diabetes: A Cross‐Sectional Study

**DOI:** 10.1111/1753-0407.70124

**Published:** 2025-07-14

**Authors:** Ruixue Feng, Donge Yan, Mingxin Bai, Xingwu Ran, Dawei Chen, Chun Wang, Lihong Chen, Shuang Lin, Sen He, Yan Liu, Murong Wu, Zhiyi Lei, Yun Gao

**Affiliations:** ^1^ Diabetic Foot Care Center, Department of Endocrinology and Metabolism West China Hospital, Sichuan University Chengdu China; ^2^ Department of Cardiology West China Hospital, Sichuan University Chengdu China; ^3^ Department of Endocrinology Chengdu Eighth People's Hospital Chengdu China; ^4^ West China Medical School Sichuan University Chengdu China

**Keywords:** cardiac autonomic dysfunction, diabetic neurogenic bladder, gastrointestinal autonomic neuropathy, left ventricular diastolic dysfunction, type 2 diabetes mellitus

## Abstract

**Background:**

To investigate the association between different categories of diabetic autonomic neuropathy (DAN) and left ventricular diastolic dysfunction (LVDD) in patients with type 2 diabetes mellitus (T2DM).

**Methods:**

We conducted a cross‐sectional study of 3440 participants with T2DM recruited in Diabetic Foot Care Center of West China Hospital, Sichuan University from January 2016 to February 2024. LVDD was assessed via echocardiography, defined as an average E/e′ ratio > 14. Twenty‐four hour Holter ECG, postvoid residual volume (PVR) examinations, and gastric emptying scintigraphy were employed to evaluate cardiac autonomic dysfunction, diabetic neurogenic bladder (DNB), and diabetic gastrointestinal autonomic neuropathy (DGAN), respectively. Logistic regression and propensity score matching (PSM) analyses were employed to examine the associations.

**Results:**

Severe cardiac autonomic dysfunction (SDNN < 50 ms) was independently associated with LVDD, with odds ratios (OR) 1.731 (95% CI: 1.103–2.719, *p* = 0,018) after adjustment for potential confounding factors. LVDD tended to be independently associated with DNB (OR 1.356; 95% CI [0.992, 1.856]; *p* = 0.056). PSM analysis further validated the independent associations of SDNN < 50 ms (OR 1.587, 95% CI 1.028, 2.450, *p* = 0.037) and DNB (OR 1.454, 95% CI 1.011, 2.090, *p* = 0.043). However, DGAN was not independently associated with LVDD. Additionally, women had a higher risk of LVDD compared to men (OR 1.995, 95% CI 1.379, 2.885, *p* < 0.001).

**Conclusions:**

Severe (SDNN < 50 ms), rather than mild–moderate, cardiac autonomic dysfunction, and DNB are independently associated with LVDD in individuals with T2DM. Additionally, women have a higher risk of LVDD than men.

## Graphical abstract

Severe cardiac autonomic dysfunction (SDNN<50 ms), and diabetic neurogenic bladder are independently associated with LVDD in individuals with T2DM.

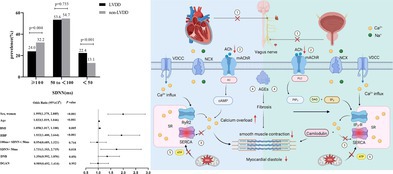

**Summary**

- Only severe cardiac autonomic dysfunction rather than those mild‐moderate cases was independently associated with LVDD.
- Interestingly, we also observed an independent association between DNB and LVDD in individuals with T2DM, which has not been reported in previous studies.

## 1 Introduction

Clinical and epidemiological studies have confirmed that individuals with diabetes mellitus (DM) have a 2‐to‐4‐fold higher risk for heart failure (HF) than those without diabetes [1]. It is still the case even in the absence of ischemic, hypertensive, or valvular heart disease [2]. Chronic hyperglycemia mediated physiological alteration and metabolic distortion can lead not only to heart failure with reduced ejection fraction (HFrEF) associated with coronary artery disease (CAD) [2], but also to diabetic cardiomyopathy without significant epicardial CAD with subsequent incident heart failure with preserved ejection fraction (HFpEF) [3].

HFpEF accounts for 50% of HF cases [4, 5], making it the most common type of HF, and its hospitalization and mortality rates are comparable to those of HFrEF [6, 7, 8]. However, HFpEF is a highly heterogeneous group of disorders with impaired left ventricular (LV) diastolic function and is difficult to treat [9]. Many treatment options for HFpEF did not respond well based on the data of clinical trials [10]. Therefore, prevention and early detection are particularly important in the management of HFpEF.

People with potential risk of developing HFpEF often experience a stage without symptoms of HF but with structural heart disease, namely asymptomatic left ventricular diastolic dysfunction (LVDD), prior to symptomatic HF [11, 12]. Although diastolic dysfunction alone is essentially part of normal human aging, asymptomatic LVDD has been shown to be an independent predictor of later development of HFpEF [13]. Apart from those recognized risk factors such as advanced age, hypertension, obesity, and CAD, diabetic autonomic neuropathy (DAN) may play a role in the development of LVDD [14]. In clinical practice, DAN may affect cardiovascular, gastrointestinal (GI), and urogenital systems, and sudomotor function.

However, there were few reports about the association of LVDD and DAN other than diabetic cardiac autonomic neuropathy (CAN). Although pathogenic mechanisms are shared across DANs, the temporal progression and clinical severity profiles of different types of DAN vary among individuals with type 2 diabetes mellitus (T2DM) [15]. Although CAN is established as an independent risk marker for LVDD, potential associations between LVDD and other DAN categories remain unevaluated.

Therefore, the present study aimed to investigate the association between various categories of DAN and LVDD in individuals with T2DM, in order to establish a basis for finding possible predictors of HFpEF in the following prospective cohort study.

## 2 Materials and Methods

### 2.1 Study Population

**FIGURE 1:**
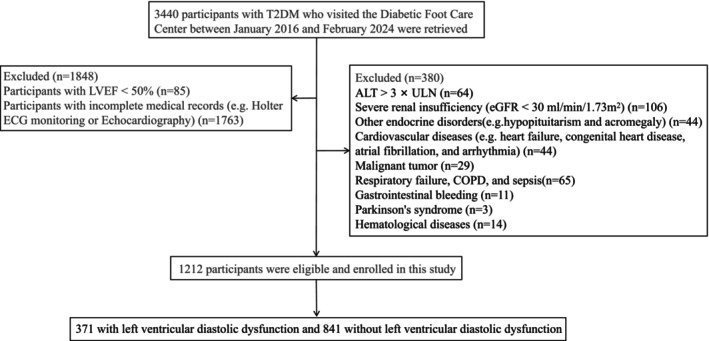
Flow chart of the study. ALT, alanine transferase; COPD, chronic obstructive pulmonary disease; ECG, electrocardiogram; eGFR, estimated glomerular filtration rate; LVEF, left ventricular ejection fraction; T2DM, type 2 diabetes mellitus; ULN, the upper limit of normal.

Between January 2016 and February 2024, 3440 participants with T2DM were recruited from the Diabetic Foot Care Center of West China Hospital, Sichuan University, Chengdu, China. The diagnosis of DM was based on the diagnostic criteria proposed by WHO in 1999 [16]. Exclusion criteria were as follows: patients with type 1 or specific types of DM, severe cardiovascular diseases such as myocardial infarction and arrhythmias, severe renal and hepatic insufficiency, other endocrine and metabolic disorders, hematologic, autoimmune, and/or rheumatologic disorders, malignant neoplasms, hemorrhage, neurologic, respiratory disorders, and severe infectious diseases. In total, 2228 participants were excluded from the present study due to incomplete information on medical records (missing data primarily from Holter ECG monitoring and Echocardiography) (*n* = 1763), LVEF < 50% (*n* = 85), and fulfillment of exclusion criteria (*n* = 380). According to these criteria, 1212 patients with T2DM (371 with LVDD and 841 non‐LVDD) were ultimately included in the study (Figure 1). The study was reviewed and approved by the Institutional Ethics Committee of the West China Hospital and was registered in the Clinical Trial Registry (registration number: ChiCTR2300076628). Written informed consent was obtained from all participants.

### 2.2 Assessment of LVDD

Transthoracic echocardiography is the most widely used noninvasive technique for evaluating cardiac structure and function. For accurate results, all participants conducted their echocardiograms after their clinical condition stabilized. All Participants underwent standard transthoracic echocardiography by an experienced echocardiographer from West China Hospital using Philips iE33 or EPIQ7 (Phillips Medical Systems, Andover, MA) ultrasound system equipped with a S5‐1 1.3–4.2 mHz transducer. Participants were placed in the left supine position, and the left atrium (LA), LV, interventricular septum (IVS), and left ventricular posterior wall (LVPW) were measured using the standard parasternal long‐axis views. Left ventricular mass (LVM) was calculated using the Devereux formula: LVM (g) = 0.8 × 1.04 × [(LVIDd + IVS + LVPW)3 − LVIDd3] + 0.6 and indexed per square meter of body surface area (LVMI) [17]. Mitral peak E velocity (E) were recorded with pulsed‐wave Doppler, and tissue Doppler‐derived mitral annular e′ velocity was acquired at septal and lateral annulus and obtained its average(e′), average E/e′ ratio was E divided by e′ Peak tricuspid regurgitation velocity (TRVmax) obtained using continuous wave doppler, the above data were obtained in the apical four‐chamber view. Echocardiographic analyses were performed independently by echocardiographers who were blinded to the participants' DAN status.

According to the standards of the American Society of Echocardiography and the European Association of Cardiovascular Imaging (ASE/EACVI), LV diastolic dysfunction was defined as an average E/e′ ratio > 14 [18].

### 2.3 Assessment of Different Types of DAN

In the current study, cardiac autonomic dysfunction, diabetic neurogenic bladder (DNB), and GI autonomic neuropathy were assessed. The cardiac autonomic dysfunction, which was defined as decreased heart rate variability (HRV), was an early manifestation of CAN [19, 20]. The details were as follows:

#### 2.3.1 Cardiac Autonomic Dysfunction

A 24‐h Holter ECG was monitored using a GE‐Marquette MARS PC ambulatory ECG Holter system. Professionals at the ECG unit of West China Hospital of Sichuan University performed the procedure. First, participants needed to be familiar with the device, the staff, and the procedures and were requested to avoid alcohol and stimulants, such as caffeine, nicotine, and chocolate, and to avoid strenuous physical activity prior to and for 24 h after recording so as to obtain reliable HRV data.

Each participant then underwent a 24‐h ambulatory electrocardiogram at the same time (8 a.m.–9 a.m.) to avoid circadian rhythm bias. Electrode pads were placed in the precordial region, and the skin should be kept dry and away from electromagnetic radiation for accurate recording of ECG activity. A Holter Analysis Workstation (GE Medical Systems Information Technologies Inc., TX, USA) processed the recorded ECG data. Time‐domain HRV indices were generated, including the standard deviation of the normal sinus interval (SDNN), the root mean square of successive RR interval differences (rMSSD), the standard deviation of the 5‐min average RR intervals (SDANN), and the percentage of normal adjacent RR interval difference > 50 ms (PNN50). The low frequency power (LF), high frequency power (HF), and LF/HF ratio are indexes of the frequency domain.

Within the indices of HRV, SDNN reflects the overall cardiac autonomic regulation and serves to assess the overall damage and recovery of the cardiac autonomic nerve [19]. In the current study, SDNN < 50 ms was defined as a highly depressed HRV [19], which reflected severe cardiac autonomic dysfunction (SDNN) [20], while 50 ≤ SDNN < 100 ms was defined as a moderately depressed HRV [19], which reflected mild–moderate cardiac autonomic dysfunction [20].

#### 2.3.2 DNB

All participants underwent Postvoid Residual Volume (PVR) examinations in the ultrasound department. Participants received an appropriate amount of water (usually around 300—500 mL) about 30 min to 1 h before the measurement to ensure that the bladder was adequately distended for accurate assessment. Participants lay in a supine position on the examination table with the lower abdomen exposed, turned on the ultrasound machine, applied a sufficient amount of ultrasound gel on the patient's lower abdomen over the bladder area, gently placed the transducer on the gel‐covered skin, and moved it around to locate the bladder in both longitudinal and transverse planes. Try to obtain a clear cross‐sectional view of the bladder that shows its full shape and outline. Calculate the PVR using the formula: PVR (mL) = 0.52 × length (cm) × width (cm) × height (cm). Record the calculated PVR value accurately. After instructing participants to completely empty their bladder, the aforementioned examination was repeated and the postvoid residual urine volume was calculated. When they presented with nocturia, frequent urination, a weak urinary stream, and/or a feeling that the bladder could not be emptied completely, participants with the volume of residual urine in their bladder for more than 10 mL were diagnosed as DNB after excluding obstructive urinary retention, central nervous system diseases, urinary tract infection, and drug‐induced urinary retention [19, 20, 21].

#### 2.3.3 GI Autonomic Neuropathy

All participants underwent gastric emptying scintigraphy (GES) which was performed in the nuclear medicine department using the Symbia Evo Excel imaging device. A dose of 2 mCi of 99mTc‐DTPA was dissolved in 200 mL of drinking water and orally ingested by the participants. Immediately following ingestion, the participants were positioned supine for dynamic gastric imaging. The images were taken using a 256 × 256 matrix with 1× magnification and a frame rate of 1 frame per minute. A total of 30 consecutive frames were acquired during the dynamic imaging phase. Subsequently, after a 30‐min interval, a delayed image was obtained at 1 h postingestion, with one frame captured at that time. Scintigraphy is considered the gold standard for assessing gastric emptying. Delayed gastric emptying was present if gastric retention was > 60% at 2 h and/or > 10% at 4 h [22]. Gastroparesis is a more severe form of delayed gastric emptying, which is often associated with significant symptoms and complications. Exclusion of reversible/iatrogenic causes such as medications or organic causes of gastric outlet obstruction or peptic ulcer disease (with esophagogas‐troduodenoscopy or a barium study of the stomach) is needed before considering a diagnosis of or specialized testing for gastroparesis. The diagnostic gold standard for gastroparesis is the measurement of gastric emptying with scintigraphy of digestible solids at 15‐min intervals for 4 h after food intake. Patients presenting nausea, vomiting, early satiety, and bloating were evaluated using GES. In the current study, the diagnosis of diabetic gastrointestinal autonomic neuropathy (DGAN) was based on a combination of patient‐reported symptoms and delayed gastric emptying or gastroparesis [23, 24].

### 2.4 Assessment of Covariates

In the study, we collected information on covariates including demographic characteristics, lifestyle risk factors, biochemical indices, and co‐morbidity history. PAD was defined by the presence of ankle brachial index (ABI) < 0.9, confirmed imaging examination of severe stenosis or occlusion of arteries in the lower extremities, or history of revascularization therapy, as previously described [25].

Diabetic peripheral neuropathy (DPN) was diagnosed based on the combination of neuropathy symptoms, signs, and neurophysiologic test abnormalities as previously reported [26]. CAD and cerebrovascular disease (CVD) were abstracted from the clinical notes of the patient. The diagnosis of diabetic retinopathy (DR) was documented by an ophthalmologist. High blood pressure (HBP) was defined as a systolic blood pressure (SBP) of 140 mmHg or greater, a diastolic BP of 90 mmHg or greater, a history of physician‐diagnosed hypertension, or self‐reported current treatment for hypertension with antihypertensive medication. Biochemical indices (e.g., eGFR, TC, TG, LDL‐C, HDL‐C, HbA1c) were measured by standard laboratory methods at the central laboratory of West China Hospital, Sichuan University.

### 2.5 Statistical Analysis

Statistical analysis was performed using SAS 9.4 software (SAS Institute Inc., Cary, NC) and R (RStudio, PBC, Boston, Massachusetts, USA). Continuous variables were expressed as mean ± SD or medians with interquartile ranges. Between‐group differences were tested for significance using Student's *t* test or Wilcoxon rank test. Categorical variables were reported as numbers and percentages, and differences were compared using Chi‐square test.

Logistic regression was used to calculate odds ratios (ORs) and 95% CIs for the association between various categories of DAN and LVDD. In the multivariable models, potential confounders were adjusted that could be associated with DAN and LVDD, including age, sex, duration of diabetes, BMI, drinking(never, occasional and frequent level), current smoking, mean blood pressure (MBP), history of HBP, CAD, CVD, PAD, DPN, DGAN, DNB, DR, eGFR, TC, TG, and HbA1c. VIFs were calculated to assess multicollinearity, and variables with VIF ≥ 5 were excluded from the final model.

Additionally, a propensity score matching (PSM) analysis was performed with the use of R software, version 4.1.0 (R Project for Statistical Computing). Individuals with LVDD were matched 1:1 with controls with non‐LVDD using the nearest neighbor caliper width of 0.05. The absolute standardized differences (ASD) for each covariate were calculated before and after PSM (an ASD ≥ 0.10 indicates imbalance). This matching procedure was performed using the MatchIt package for R (RStudio, PBC, Boston, Massachusetts, USA). Additionally, univariate and multivariate regression analyses were also carried out after PSM. Statistical significance was accepted as a two‐sided test with an alpha level of 0.05.

## 3 Results

The baseline demographic and clinical characteristics of all participants are shown in Table 1. Individuals with LVDD were likely to be women and older, had a longer duration of diabetes, and had higher proportions of tobacco and alcohol consumption. In addition, individuals with LVDD were likely to have a higher BMI, SBP, and MBP, lower HbA1c and eGFR, and to be at a higher risk of having HBP, CAD, DPN, PAD, DNB, DR, CKD than those with non‐LVDD.

**TABLE 1:** Demographic and clinical characteristics at baseline.

Variables	LVDD group	Non‐LVDD group	*t*/*χ* ^2^	*p*
No. of patients (*n*)	371	841		
Sex, men (%)	183 (49.3)	575 (68.4)	39.857	< 0.001
Age (year)	66.8 ± 11.9	58.7 ± 14.0	−10.278	< 0.001
Diabetes duration (year)	13 (7, 20)	10 (4, 16)	−5.220	< 0.001
Drinking history, *n* (%)			10.597	0.005
Never	262 (70.6)	516 (61.4)		
Occasional	42 (11.3)	144 (17.1)		
Frequent	67 (18.1)	181 (21.5)		
Smoking history, *n* (%)	117 (31.5)	381 (45.3)	20.156	< 0.001
BMI (kg/m^2^)	24.4 ± 3.8	23.8 ± 3.8	−2.379	0.017
SBP (mmHg)	145.3 ± 20.5	135.1 ± 21.1	−7.851	< 0.001
DBP (mmHg)	81.6 ± 13.0	82.7 ± 12.0	1.314	0.189
MBP (mmHg)	102.8 ± 13.3	100.1 ± 13.7	−3.277	0.001
HbA1c (%)	8.2 (7.2, 9.7)	8.5 (7.1, 10.7)	3.089	0.036
FPG (mmol/L)	8.3 (6.3, 10.5)	8.5 (6.5, 11.5)	1.775	0.057
TC (mmol/L)	4.06 ± 1.24	4.13 ± 1.27	0.873	0.383
TG (mmol/L)	1.42 (1.04, 2.01)	1.38 (0.98, 2.09)	1.274	0.576
HDL‐C (mmol/L)	1.10 ± 0.37	1.11 ± 0.40	0.174	0.860
LDL‐C (mmol/L)	2.44 ± 0.99	2.43 ± 0.98	−0.227	0.821
eGFR (mL/min/1.73m^2^)	76.0 ± 23.9	87.7 ± 24.5	7.838	< 0.001
HBP, *n*(%)	281 (75.7)	418 (49.7)	71.501	< 0.001
CAD, *n*(%)	83 (22.4)	98 (11.7)	23.284	< 0.001
CVD, *n*(%)	27 (7.3)	42 (5.0)	2.500	0.114
DPN, *n*(%)	309 (83.3)	604 (71.8)	18.222	< 0.001
PAD, *n*(%)	127 (34.2)	160 (19.0)	32.940	< 0.001
DGAN, n(%)	75 (20.2)	150 (17.8)	0.964	0.326
DNB, *n*(%)	133 (35.8)	201 (23.9)	18.412	< 0.001
DR, *n*(%)	146 (39.4)	272 (32.3)	5.600	0.018
CKD, *n*(%)	114 (30.7)	144 (17.1)	20.912	< 0.001
CAN, *n*(%)	222 (59.8)	453 (53.9)	3.723	0.054

Abbreviations: BMI, body mass index; CAD, coronary artery disease; CAN, cardiac autonomic neuropathy; CKD, chronic kidney disease; CVD, cerebrovascular disease; DBP, diastolic blood pressure; DGAN, diabetic gastrointestinal autonomic neuropathy; DNB, diabetic neurogenic bladder; DPN, diabetic peripheral neuropathy; DR, diabetic retinopathy; eGFR, estimated glomerular filtration rate; FPG, fasting plasma glucose; HbA1c, glycated hemoglobin; HBP, hypertension; HDL‐C, high‐density lipoprotein cholesterol; LDL‐C, low‐density lipoprotein cholesterol; LVDD, left ventricular diastolic dysfunction; MBP, mean blood pressure; PAD, peripheral arterial disease; SBP, systolic blood pressure; TC, total cholesterol; TG, triglycerides.

Indices of cardiac structure, diastolic function, and HRV are shown in Table 2. Compared with those with non‐LVDD, individuals with LVDD had larger LA, thicker LVPW, and thicker IVS (all *p* < 0.001), suggesting reduced cardiac compliance. Additionally, SDNN and LF/HF were significantly lower in the LVDD group compared with the non‐LVDD group (all *p* < 0.001). SDANN and LF were significantly lower in the LVDD group than in the non‐LVDD group (all *p* < 0.001). There was no significant difference in PNN50, rMSSD, and HF between the two groups.

**TABLE 2:** Cardiac structure, diastolic function and heart rate variability in participants with and without LVDD.

Variables	LVDD group	Non‐LVDD group	*t*/*χ* ^2^	*p*
No. of patients (*n*)	371	841		
LA (mm)	35.47 ± 5.35	33.05 ± 4.02	−7.784	< 0.001
LVPW (mm)	9.75 ± 1.40	9.34 ± 1.46	−4.663	< 0.001
IVS (mm)	11.51 ± 1.84	10.74 ± 1.83	−6.724	< 0.001
LV (mm)	45.86 ± 4.05	45.84 ± 3.58	−0.090	0.929
RV (mm)	21.04 ± 2.40	20.90 ± 2.29	−0.978	0.328
RA (mm)	32.77 ± 4.21	32.53 ± 3.85	−0.915	0.364
LVEF (%)	67.25 ± 5.21	67.14 ± 5.05	−0.343	0.728
EDV (mL)	97.76 ± 21.20	97.12 ± 18.88	−0.499	0.617
ESV(ml)	32.35 ± 10.37	32.62 ± 12.85	0.381	0.705
SV (mL)	65.38 ± 13.46	65.18 ± 12.37	−0.239	0.811
E (m/s)	0.80 (0.70, 1.00)	0.70 (0.60, 0.80)	−4.443	< 0.001
e′ (cm/s)	4.00 (4.00, 5.00)	6.00 (5.00, 7.70)	21.746	< 0.001
E/e′	17.9 (15.0, 20.0)	10.0 (9.0, 12.0)	0.000	< 0.001
HR (bpm)	77.1 ± 10.9	78.9 ± 10.6	2.625	0.009
SDNN (ms)	75 (53, 97)	84 (60, 109)	4.094	< 0.001
LF/HF	1.2 ± 0.5	1.4 ± 0.5	6.787	< 0.001
HF (ms^2^)	5.7 (3.9, 7.6)	6.2 (4.1, 8.8)	2.914	0.013
PNN50 (%)	0.8 (0.1, 4.1)	1.1 (0.1, 4.3)	1.221	0.133
rMSSD (ms)	15.0 (11.0, 23.0)	17.0 (11.0, 23.0)	1.827	0.127
SDANN (ms)	69.1 ± 28.8	80.6 ± 42.5	5.460	< 0.001
LF (ms^2^)	6.9 (3.8, 9.9)	8.9 (5.0, 13.6)	6.166	< 0.001

Abbreviations: E, the peak early diastolic mitral inflow velocity; e′, the mitral annulus early diastolic velocity; EDV, end‐diastolic volume; ESV, end‐systolic volume; HF, high frequency power; HR, heart rate; IVS, interventricular septum; LA, left atrium; LF, low frequency power; LF/HF, rate of low frequency power between high frequency power; LV, left ventricle; LVDD, left ventricular diastolic dysfunction; LVEF, left ventricular ejection fractions; LVPW, left ventricular posterior wall; PNN50, the percentage of normal adjacent RR interval difference > 50 ms; RA, right atrium; rMSSD, the root mean square of successive RR interval differences; RV, right ventricle; SDANN, the standard deviation of the 5 min average RR intervals; SDNN, the standard deviation of normal sinus interval; SV, stroke volume.

**FIGURE 2:**
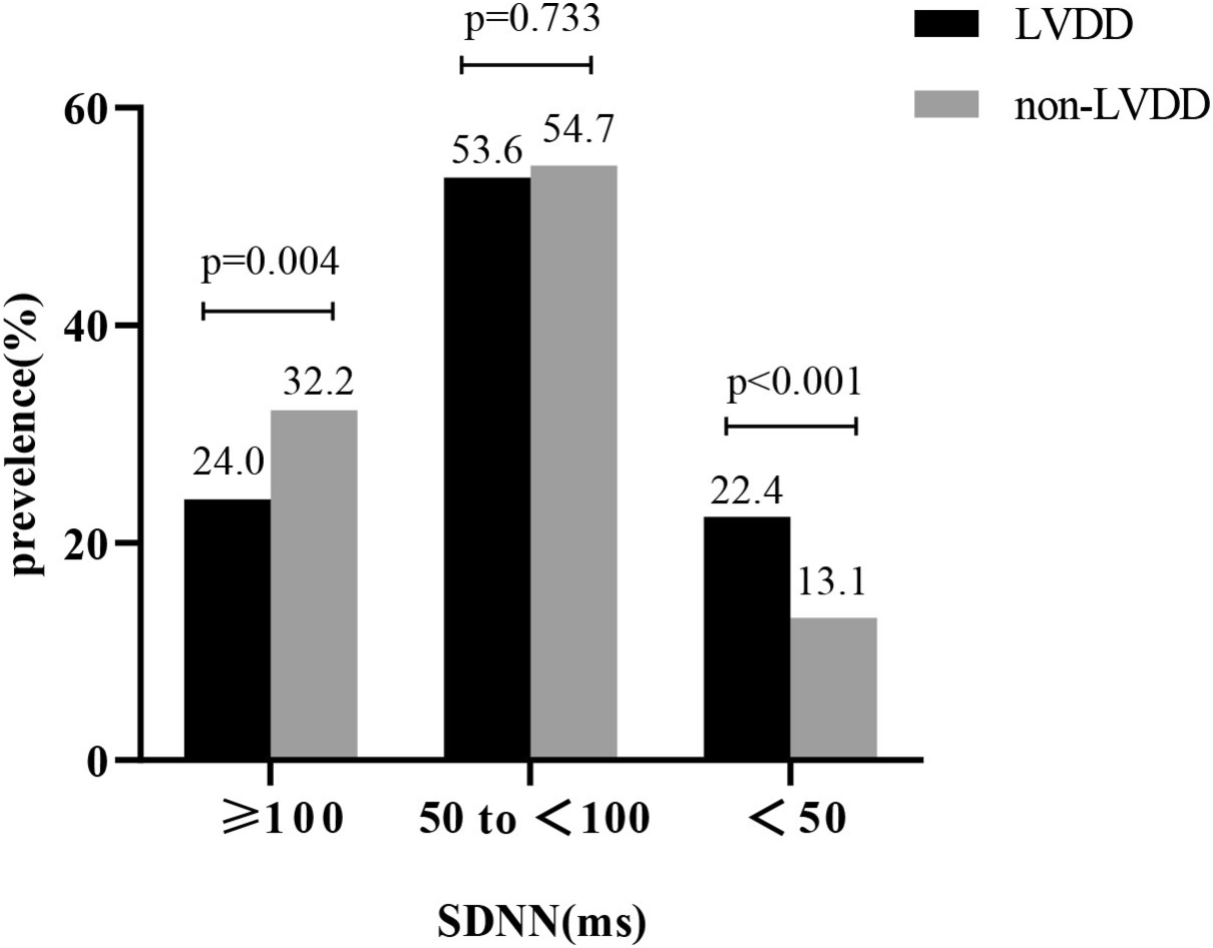
The prevalence and severity of cardiac autonomic dysfunction in participants with and without left ventricular diastolic dysfunction. LVDD, left ventricular diastolic dysfunction; SDNN, the standard deviation of normal sinus interval.

If the severity of cardiac autonomic dysfunction was stratified based on decreasing level of SDNN, individuals with LVDD had a 1.7‐fold increase in SDNN (i.e., SDNN < 50 ms) (22.4% vs. 13.1%, *p* < 0.001) than those with non‐LVDD.(Figure 2).

**FIGURE 3:**
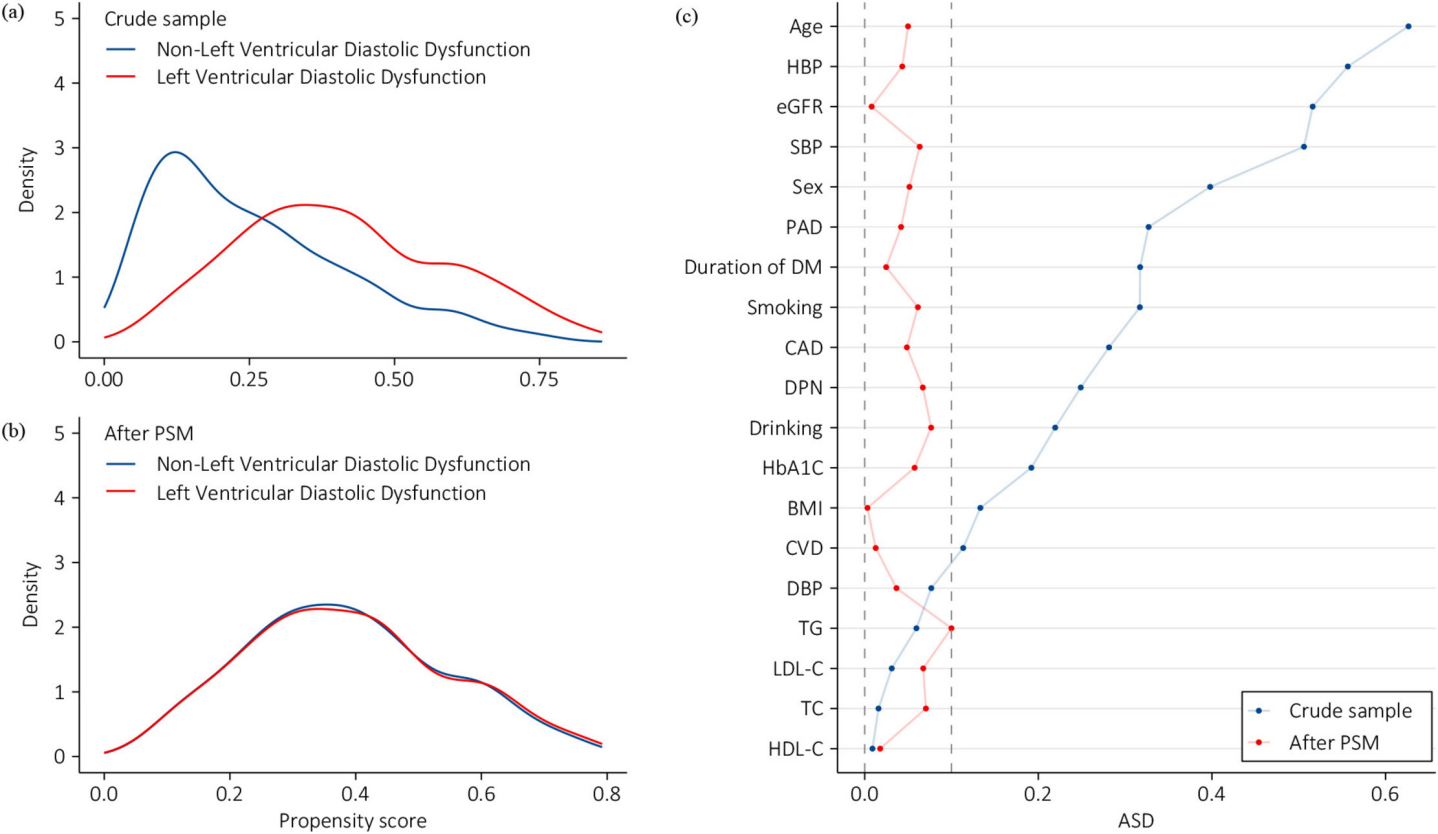
Logistic regression analysis of factors associated with left ventricular diastolic dysfunction in participants with T2DM. Adjustment for imbalanced variables, including diabetic duration, MBP, smoking history, drinking history, eGFR, TC, TG, HbA1C, PAD, CAD, CVD, DPN, DGAN, DR. BMI, body mass index; CAD, coronary artery disease; CVD, cerebrovascular disease; DGAN, diabetic gastrointestinal autonomic neuropathy; DNB, diabetic neurogenic bladder; DPN, diabetic peripheral neuropathy; DR, diabetic retinopathy; eGFR, estimated glomerular filtration rate; HbA1c, glycated hemoglobin; HBP, hypertension; MBP, mean blood pressure; PAD, peripheral arterial disease; PSM, propensity score matching; SDNN, the standard deviation of normal sinus interval; T2DM, type 2 diabetes mellitus; TC, total cholesterol; TG, triglycerides.

For all enrolled participants, multivariate logistic regression analysis showed that LVDD was independently and positively associated with the following risk factors: women (OR 1.995; 95% CI [1.379, 2.885]; *p* < 0.001); age (OR 1.033; 95% CI [1.019, 1.046]; *p* < 0.001); BMI (OR 1.058; 95% CI [1.017, 1.100]; *p* = 0.005); HBP (OR 1.932; 95% CI [1.400, 2.664]; *p* < 0.001); SDNN < 50 ms (OR 1.731; 95% CI [1.103, 2.719]; *p* = 0.018), adjusting for other potential confounders. LVDD tended to be independently associated with DNB (OR 1.356; 95% CI [0.992, 1.856]; *p* = 0.056) (Figure 3). However, there was no independent association between LVDD and DGAN (OR 0.989; 95% CI [0.692, 1.414]; *p* = 0.952).

**FIGURE 4:**
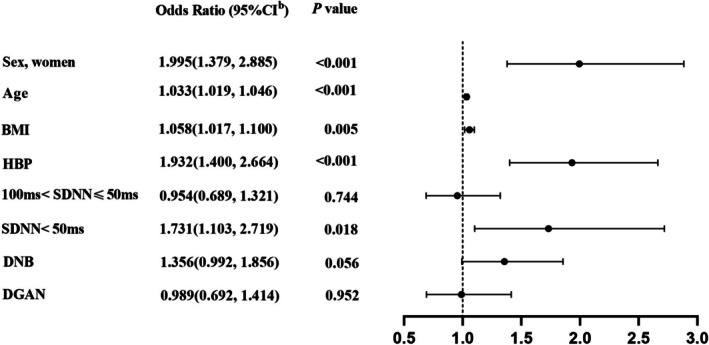
Propensity scores (PS) distributional overlap and absolute standardized differences (ASD) in participants with and without left ventricular diastolic dysfunction (LVDD). (a and b) Present PS distributions between individuals with and without LVDD in the crude sample and the sample after propensity score matching (PSM). For intervals along the x‐axis, the area under the probability density curve represents the probability of those PSs, and smoothing was through the kernel density estimate. The higher the overlap of the two sets of PS curves, the lower the risk of confounding. (c) Dotplot of absolute standardized differences before and after matching. The dashed line indicates > 0.1 imbalance between the variable's value, which is a commonly used metric of significant imbalance. BMI, body mass index; CAD, coronary artery disease; CVD, cerebrovascular disease; DBP, diastolic blood pressure; DM, diabetes mellitus; DPN, diabetic peripheral neuropathy; eGFR, estimated glomerular filtration rate; HbA1c, glycated hemoglobin; HDL‐C, high‐density lipoprotein cholesterol; LDL‐C, low‐density lipoprotein cholesterol; PAD, peripheral arterial disease; SBP, systolic blood pressure; TC, total cholesterol; TG, triglycerides.

In order to better balance covariates between the two groups, PSM was employed to verify the association of LVDD and cardiac autonomic dysfunction as well as DNB. Figure 4a showed the distribution of propensity scores (PS) in individuals with LVDD and with non‐LVDD before PSM. The smaller the overlap between the PS curves of the two groups was, the greater the risk of confounder was. After PSM, the PS curves between the group with LVDD and the counterparts with non‐LVDD were highly overlapping, suggesting a good balance of baseline covariates between the two groups (Figure 4b). Figure 4c shows the ASD before and after PSM. Significant imbalances can be ruled out as all covariates in PS have ASDs below 0.10 after PSM. In the PSM analysis, SDNN < 50 ms (OR 1.587; 95% CI [1.028, 2.450]; *p* = 0.037) remained an independent association of LVDD. Moreover, the independent association of DNB with higher risk of LVDD reached statistical significance (OR 1.454; 95% CI [1.011, 2.090]; *p* = 0.043) (Table 3).

**TABLE 3:** Logistic regression model of factors associated with left ventricular diastolic dysfunction in participants with T2DM after PSM.

	Model 1		Model 2	
	ORs (95% CI)	*p*	ORs (95% CI)	*p*
Sex, women	1.126 (0.832, 1.523)	0.441	1.142 (0.749, 1.740)	0.538
Age	0.999 (0.986, 1.012)	0.879	0.997 (0.982, 1.013)	0.734
BMI	0.994 (0.954, 1.036)	0.769	0.992 (0.948, 1.038)	0.722
HBP	1.030 (0.734, 1.447)	0.862	0.989 (0.679, 1.439)	0.952
SDNN < 50 ms	1.670 (1.126, 2.476)	0.011	1.587 (1.028, 2.450)	0.037
DNB	1.489 (1.075, 2.064)	0.017	1.454 (1.011, 2.090)	0.043
DGAN	1.038 (0.711, 1.515)	0.847	0.910 (0.606, 1.365)	0.649

*Note:* All values are adjusted mean differences (95% CIs). Model 1 was the univariate logistic regression model. Model 2 was the multivariate logistic regression model with adjustment for imbalance diabetic duration, MBP, smoking history, drinking history, eGFR, TC, TG, HbA1C, PAD, CAD, CVD, DPN, DGAN, DR.

Abbreviations: BMI, body mass index; CAD, coronary artery disease; CVD, cerebrovascular disease; DGAN, diabetic gastrointestinal autonomic neuropathy; DNB, diabetic neurogenic bladder; DPN, diabetic peripheral neuropathy; DR, diabetic retinopathy; eGFR, estimated glomerular filtration rate; HbA1c, glycated hemoglobin; HBP, hypertension; MBP, mean blood pressure; PAD, peripheral arterial disease; PSM, propensity score matching; SDNN, the standard deviation of normal sinus interval; T2DM, type 2 diabetes mellitus; TC, total cholesterol; TG, triglycerides.

**FIGURE 5:**
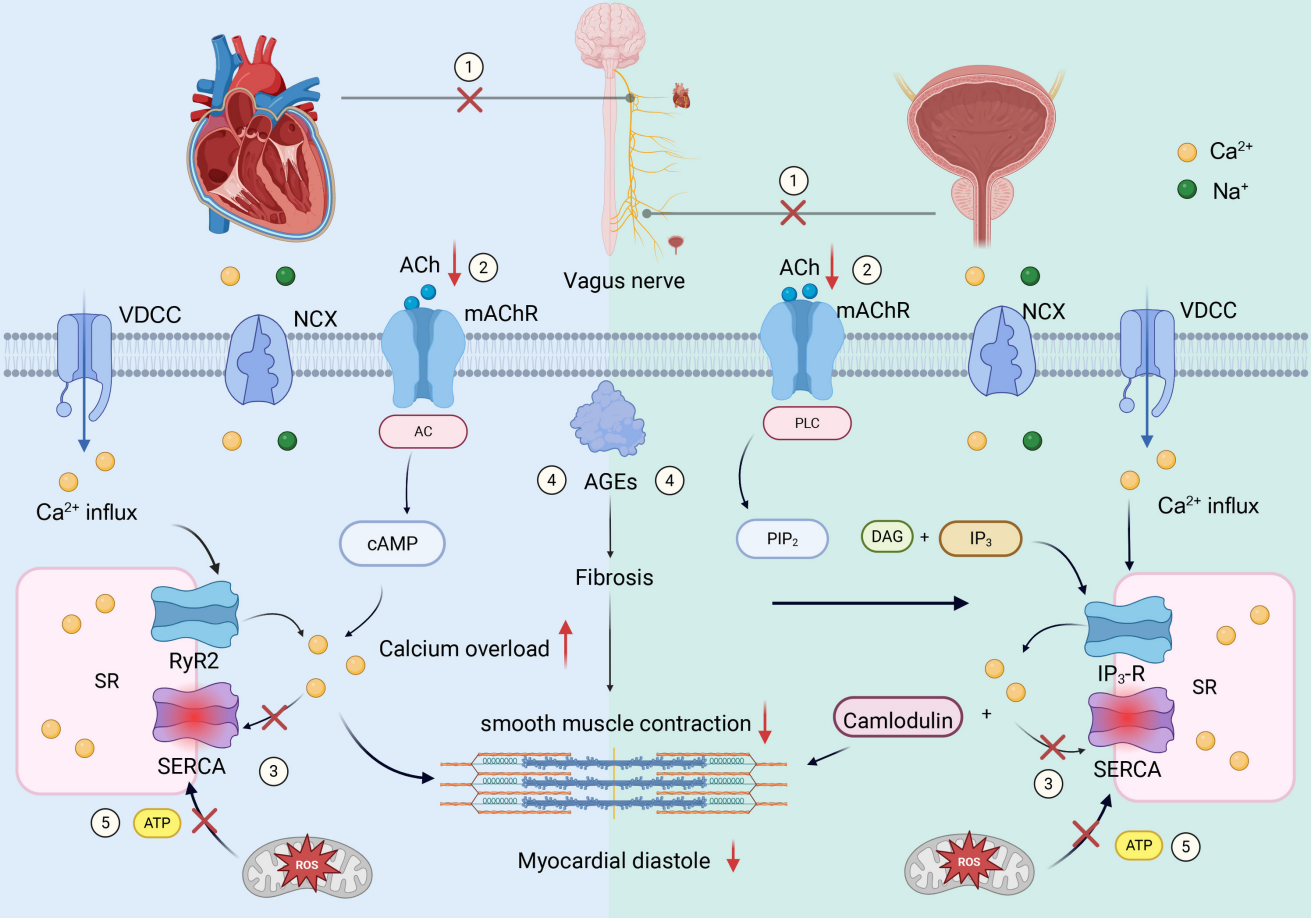
Pathophysiologic mechanisms underlying the relationship between DNB and LVDD in T2DM. mAchR, muscarinic acetylcholine receptor; NCX, Na^+^/Ca^2+^ exchangers; RyR2, ryanodine receptor; SERCA, sarcoendoplasmic reticulum calcium ATPase; VDCC, voltage‐gated calcium channel.

## 4 Discussion

The current study investigated the association between various categories of DAN and LVDD in individuals with T2DM. We found that SDNN < 50 ms in the HRV index and DNB were independently associated with LVDD, after adjustment for potential confounding factors such as advanced age, hypertension, overweight, and other chronic conditions. The PS analysis showed a consistent result. Additionally, women had a higher risk of LVDD than men after adjustment for confounding factors. However, GI autonomic neuropathy was not independently associated with LVDD.

Previous studies have been generally consistent in reporting that CAN was associated with LVDD [27, 28]. Specifically, CAN in diabetes contributes to LVDD by increasing sympathetic tone, which impairs myocardial relaxation and ventricular filling during diastole, leading to diastolic stiffness and dysfunction of the left ventricle [29]. The underlying mechanisms driving this phenomenon include altered calcium handling in cardiomyocytes and extracellular matrix remodeling, both of which are exacerbated by the metabolic disturbances associated with diabetes [30]. Furthermore, elevated circulating glucose levels and the accumulation of advanced glycation end‐products (AGEs) significantly contribute to myocardial stiffness by promoting collagen deposition and crosslinking [31]. In clinical practice, CAN is usually documented by using several cardiovascular autonomic reflex tests (CARTs). However, CARTs often fail to detect CAN at an early stage. Currently, HRV is the most sensitive indicator for assessing the cardiac autonomic nervous system. Although it is not recommended by guidelines as a diagnostic criterion for CAN, decreased HRV has predictive value for adverse cardiovascular events and mortality among people with DM [32]. Moreover, decreased HRV does reflect cardiac autonomic dysregulation, which may be an early suggestion of CAN. SDNN is the most commonly used time domain measure of HRV and a widely accepted marker of total tension of sympathetic and vagal nerves. Jus Ksela et al. reported that decreased values of SDNN were associated with worse 1‐year prognosis in patients with HFpEF, and the extent of autonomic disbalance as determined by HRV could potentially assist in the prognostic assessment and risk stratification of patients with HFpEF [33]. Despite the significant association between decreased SDNN with LVDD or HFpEF, our study revealed that only SDNN (< 50 ms) rather than those mild–moderate cases(50 ≤ SDNN < 100 ms) was independently associated with LVDD. Thus, the different extent of decreased SDNN may have different effects on LVDD or HFpEF, which has been rarely addressed in previous studies. Then, whether decreased SDNN could serve as an early clinical predictor for symptomatic LVDD or even HFpEF needs to be confirmed in our ongoing prospective cohort study.

Interestingly, we observed an independent association between DNB and LVDD in individuals with T2DM. Moreover, the sensitivity analysis in PSM showed a consistent result. Previous studies mainly focused on the relationship between CAN and LVDD, while the association of DNB and LVDD has rarely been reported. To our knowledge, this was the first clinical study to investigate the association of DNB and LVDD. As the different categories of DAN, DNB and CAN shared the common pathological mechanisms. Current hypotheses of related mechanisms include the following: (1) The diastolic function of the heart and the detrusor muscle of the bladder are both closely linked to parasympathetic nerve activity. Due to their extended anatomical courses, parasympathetic nerves (i.e., vagus nerve and pelvic nerve) are impaired earlier and more severely affected than sympathetic nerves among individuals with T2DM. Structural damage and functional decline of parasympathetic fibers reduce ACh release, initiating downstream dysfunction in both organs; (2) reduced ACh results in impaired AC/cAMP and PLC/IP3 pathways and reduced Ca^2+^ transients and contractility in myocardium/detrusor; (3) decreased activity of SERCA2a due to oxidative stress decreased reuptake of Ca^2+^ by the sarcoplasmic reticulum, leading to cytosolic Ca^2+^ overload and impaired relaxation; (4) accumulation of advanced glycation end products (AGEs) causes myocardial and bladder fibrosis and decreased compliance; (5) ROS lead to mitochondrial dysfunction reducing ATP production, further exacerbating SERCA2a failure and Ca^2+^ dysregulation [27, 34, 35, 36] (Figure 5). In summary, the integrated mechanisms mentioned above can lead to decreased myocardial compliance during diastole and/or impaired bladder voiding function. The timing of DNB and CAN is not synchronized; instead, it is variable. In other words, individuals with CAN do not necessarily develop neurogenic bladder, and individuals with neurogenic bladder do not necessarily have severe CAN. Therefore, considering that DNB is independently associated with LVDD, it may, like CAN, also serve as a potential predictor of future cardiovascular events, which requires verification through prospective studies.

DGAN is also a common type of DAN. A delayed radionucleotide gastric emptying cohort study from the USA population identified a correlation between diabetic gastroparesis and some known risk factors of LVDD such as cardiovascular disease, hypertension, and DR [37]. By the time, however, there is no report on the direct association between DGAN and LVDD. In the present study, we also first investigated the association of DGAN and LVDD. Nevertheless, we did not observe a significant association between DGAN and LVDD. The result may be attributed to the diagnostic method of DGAN. In the present study, the diagnosis of DGAN was based on the reports of radionucleotide GES in patients with DM and symptoms suggestive of gastroparesis. However, we found the excessive sensitivity of delayed radionucleotide gastric emptying as the diagnostic criterion of DGAN in our clinical practice. Previous studies also showed that the GES had a high sensitivity for detecting delayed gastric emptying but with low specificity, leading to false positives in some cases due to overlap with other GI conditions [38, 39]. Hence, the prevalence of DGAN may have been overestimated [39]. Additionally, the severity of DGAN cannot be quantified or stratified in the present study. Thus, it is not clear whether severe DGAN rather than mild DGAN may be associated with LVDD. These questions will be further explored in our ongoing prospective cohort study.

In addition, our study showed that female patients were at a higher risk for the development of LVDD than their male counterparts. In agreement with our findings, in the Framingham Heart Study, DM was associated with a nearly two fold increase in the risk of incident HF in men and a four fold increase in women, even after adjustment for other cardiovascular risk factors [40]. This emphasizes the necessity of early cardiovascular screening in females, particularly in middle‐aged and older populations, and is critically important for the early detection of LVDD.

Our study is distinguished by its large sample size, as well as the rigorous and comprehensive inclusion and exclusion criteria, which ensured that the sample was well‐suited to address the research objectives. The study investigated the relationship between different types of DAN, respectively, particularly DNB and DGAN, with LVDD. Unexpectedly, an independent association between DNB and LVDD was discovered, which had not been reported in previous studies. Furthermore, we stratified the severity of diabetic cardiac autonomic dysfunction and found that only severe SDNN < 50 ms was associated with LVDD instead of simple SDNN, which had also drawn little attention in previous studies.

Nevertheless, several limitations must be acknowledged. First, given the cross‐sectional design of the study, causality could not be inferred from the observed associations. Second, given that the majority of the enrolled participants lacked indications for coronary angiography or CT angiography, the relevant data concerning the severity of coronary stenosis were not obtainable, which may affect cardiac autonomic function, cardiac structure, and diastolic function. Third, there may be possible misclassification in autonomic neuropathy diagnosis due to the absence of specific diagnostic tools. Finally, this study is a single‐center one, which might be prone to selection bias. Hence, further validation is required through multicenter studies.

## 5 Conclusion

Our study demonstrated that SDNN (< 50 ms) rather than mild cardiac autonomic dysfunction (50 ≤ SDNN < 100 ms) and DNB were independently associated with LVDD. However, whether these factors serve as significant predictors of future cardiovascular events such as HFpEF requires validation in prospective cohort studies. Additionally, our findings suggest that women had a higher risk of LVDD than men after adjustment for confounding factors, highlighting the need for further research into gender‐specific risk factors.

## Author Contributions

Ruixue Feng performed statistical analyses and wrote the manuscript. Donge Yan, Mingxin Bai, Yan Liu, Murong Wu, Zhiyi Lei, and Sen He helped in statistical analysis and data collection and critically reviewed the manuscript. Xingwu Ran, Dawei Chen, Chun Wang, Lihong Chen, and Shuang Lin critically reviewed and edited the manuscript. Ruixue Feng, Yun Gao, and Donge Yan conceptualized the study concept and design and edited the manuscript. All authors have read and approved the final manuscript.

## Ethics Statement

The study was reviewed and approved by the Institutional Ethics Committee of West China Hospital and has been registered in the Clinical Trial Registry (registration number: CHiCTR2300076628). All patients provided written informed consent.

## Conflicts of Interest

The authors declare no conflicts of interest.

## Data Availability

The datasets analyzed during the current study are available from the corresponding author on reasonable request.
